# Effects of inflammation and oxidative stress on postoperative delirium in cardiac surgery

**DOI:** 10.3389/fcvm.2022.1049600

**Published:** 2022-11-22

**Authors:** Yi Pang, Yuntao Li, Yonggang Zhang, Hongfa Wang, Junhui Lang, Liang Han, He Liu, Xiaoxing Xiong, Lijuan Gu, Xiaomin Wu

**Affiliations:** ^1^Bengbu Medical College, Bengbu, Anhui, China; ^2^Department of Neurosurgery, Renmin Hospital of Wuhan University, Wuhan, China; ^3^Center for Rehabilitation Medicine, Department of Anesthesiology, Zhejiang Provincial People’s Hospital, Affiliated People’s Hospital, Hangzhou Medical College, Hangzhou, Zhejiang, China; ^4^Department of Anesthesiology, The Affiliated Huzhou Hospital, Zhejiang University School of Medicine, Huzhou Central Hospital, Huzhou, China; ^5^Central Laboratory, Renmin Hospital of Wuhan University, Wuhan, China

**Keywords:** postoperative delirium, oxidative stress, inflammation, cardiopulmanory bypass, cardiac surgery

## Abstract

The past decade has witnessed unprecedented medical progress, which has translated into cardiac surgery being increasingly common and safe. However, complications such as postoperative delirium remain a major concern. Although the pathophysiological changes of delirium after cardiac surgery remain poorly understood, it is widely thought that inflammation and oxidative stress may be potential triggers of delirium. The development of delirium following cardiac surgery is associated with perioperative risk factors. Multiple interventions are being explored to prevent and treat delirium. Therefore, research on the potential role of biomarkers in delirium as well as identification of perioperative risk factors and pharmacological interventions are necessary to mitigate the development of delirium.

## Introduction

Postoperative delirium (POD) is a common acute neurocognitive disorder after cardiac surgery characterized by cognitive decline, fluctuating mental status, impaired consciousness, inattention, or confusion, resulting in severe consequences for patients (1). Although delirium occurs in patients of all ages, current evidence suggests it is most likely to occur in elderly patients with preoperative chronic central nervous system disease. It has been reported to affect 20–30% of elderly patients admitted to the hospital on an emergency basis and can result in varying degrees of adverse outcomes, including functional decline, permanent cognitive decline and mortality (2). During clinical practice, the incidence of POD in patients undergoing general surgery is 10-46%, while it can reach 50–67% in patients after cardiac surgery (3–5). In addition, the incidence of POD can reach up to 72%, depending on the type of heart surgery (6) (Table 1). POD is detrimental to neuronal activity metabolism and leads to long-term cognitive decline, functional degeneration, and poor prognosis (7, 8). An increasing body of evidence suggests that older age, preoperative cognitive impairment, type of perioperative medication administered, and preoperative conditions, including anemia, electrolyte abnormalities, dehydration and malnutrition, are risk factors for POD (9–13). Although the risk factors associated with delirium are well established, the pathogenesis of its occurrence is not well understood, thus hindering the development of effective prevention and treatment of delirium. Interestingly, it is widely thought that cardiopulmonary bypass (CPB) in cardiac surgery is a primary activator of the inflammatory response (14, 15). Previous studies reported that the potential pathogenesis of delirium might include acute central cholinergic deficiency, oxidative stress, decreased GABA-ergic activity, abnormal melatonin and serotonin pathways, noradrenergic hyperactivity, neuroinflammation leading to neuronal damage, and cerebral hypoperfusion (16–18). The purpose of this review is to discuss the pathophysiology of delirium after cardiac surgery, risk factors, and the potential role of pharmacological interventions that may reduce postoperative delirium.

**TABLE 1:** A comparison of the incidence of delirium after different cardiac procedures.

Study	Study design	Surgery type	Sample size	Age, year	Outcome measurement	Delirium assessment tool	No. of patients with POD
Li et al. (19)	Retrospective	CABG	1426	71.28 ± 4.768	ICU stay time	CAM-ICU	560 (39.3%)
Lecho wicz et al. (20)	Retrospective	CABG	1098	65.5 ± 9.8	1-year and 1month mortality; ICU stay time and hospitalization time	CAM-ICU	164 (14.9%)
Brown et al. (21)	Prospective	CABG	66	69.6 ± 7.4	Hospital mortality; Hospital days; ICU time	CAM/CAM-ICU	37 (56.1%)
Norkie ne et al. (22)	Prospective	CABG	1367	65.0 ± 9.2	Hospital mortality; ICU stay time; Mechanical ventilation time	DSM	42 (3.1%)
Liu et al. (23)	Retrospective	AD (Type- A)	100	51.90 ± 9.65	ICU stay time	CAM-ICU	34 (34%)
Cai et al. (24)	Retrospective	AD (Type- A)	301	50.66 ± 12.24	Hospital mortality; Hospital days; ICU time	CAM-ICU	73 (24.25%)
Liu et al. (25)	Retrospective	AD (Type- B)	517	53.2 ± 10.9	Hospital days; ICU stay time	CAM-ICU	69 (13.3%)
He et al. (26)	Retrospective	AD (Type- A)	438	57.89 ± 12.41	ICU stay time	CAM/CAM-ICU/RASS	78 (17.8%)
Humbe Rt et al. (27)	Prospective	SAVR	27	82.1	Hospital days	CAM	8 (30%)
Rao et al. (28)	Prospective	SAVR	77	81.3 ± 6.4	Hospital days	MMSE/CAM	39 (50.7%)
Shi et al. (29)	Prospective	SAVR	77	77.9 ± 5.3	Hospital days	CAM/CAM-S	39 (60.7%)
Wesselink et al. (30)	Retrospective	TAVR	675	77-85	Hospital days	DOS	93 (14%)
Van der Wul et al. (31)	Prospective	TAVR	703	75-84	30-days to 5years mortality	DSM	116 (16.5%)
Goudz waard et al. (32)	Prospective	TAVR	543	79.1 ± 8.0	1years mortality	DSM	75 (14%)
Körber et al. (33)	Prospective	MVR	177	72-82	ICU stay time	CAM-ICU	16 (9%)

CABG, coronary artery bypass graft; AD, aortic dissection; SAVR, surgical aortic valve replacement; TAVR, transcatheter aortic valve replacement; MVR, mitral valve repairment; ICU, intensive care unit; POD, postoperative delirium; CAM, Confusion Assessment Method; CAM-ICU, Confusion Assessment Method for ICU; MMSE, Mini-mental State Examination; DOS, Delirium Observation Scale; RASS, Rating Sedation Scale.

## Pathophysiology

**FIGURE 1:**
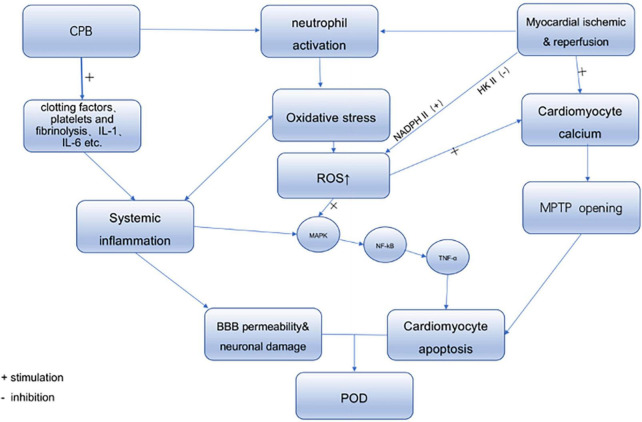
The source of inflammatory response in cardiac surgery and its effect on POD.

The underlying mechanisms behind delirium are unclear. Many hypotheses exist for the pathophysiology of delirium, such as neuroinflammation and oxidative stress. There is growing evidence that different factors, such as underlying preoperative diseases associated with inflammation, such as cardiovascular disease and diabetes, surgical trauma, extracorporeal circulation, and organ reperfusion injury during cardiac surgery, can lead to a complex inflammatory response. Therefore, we will describe the various sources of perioperative inflammation from cardiac surgery (Figure 1).

## Sources of inflammatory response in cardiac surgery

### Inflammatory state

As a result of population aging, the incidence of cardiovascular disease in the elderly has significantly increased. Nowadays, many elderly patients undergo cardiac surgery of varying complexities (34). However, cardiac surgery patients are often associated with various underlying preoperative comorbidities, such as localized regional or systemic atherosclerosis, diabetes, and pulmonary and kidney diseases, which are related to abnormal redox status and oxidative stress (35, 36). Current evidence indicates that atherosclerosis is a chronic inflammatory disease with an autoimmune component (37). In addition, it has been established that risk factors contributing to atherosclerosis, such as diabetes and dyslipidemia, promote the inflammatory response in blood vessels and indirectly increase atherosclerotic (38, 39). Although it has been shown that atherosclerotic coronary artery disease requiring intervention is related to pre-procedural oxidative stress and inflammation and is significantly exacerbated during CPB (40), this chronic disease associated with the inflammatory response has not attracted significant interest from the scientific community, probably because many of these inflammatory markers are not suitable for routine risk assessment (41).

### Systemic inflammation associated with cardiopulmonary bypass

During cardiac surgery, various factors can lead to the development of both systemic and non-systemic inflammatory responses. At the same time, injurious processes may also trigger the onset of the systemic inflammatory response during surgical trauma. Furthermore, this inflammatory response leads to synaptic damage, neuronal dysfunction and death, and neurogenesis disorders (42). The most crucial factor leading to systemic inflammatory response is CPB. It has been shown that CPB in cardiac surgery causes an intense inflammatory response through various mechanisms (14, 43), including primarily blood contact with foreign surfaces of the CPB circuit, surgical trauma and endotoxemia (44). Inflammatory responses associated with CPB include activation of coagulation factors, platelets and fibrinolysis, elevation of inflammatory cytokines including interleukin (IL)-1, IL-6, and tumor necrosis factor-α, and activation of endothelial cells and leukocyte responses (45). In addition, a previous study found that patients experiencing CPB demonstrated augmented expression of leukocyte-mRNA not only for pro-inflammatory cytokines, CAMs (i.e., platelet endothelial cell adhesion molecule-PECAM) but also for IL-10 and heme oxygenase-1 (46). In addition to CPB, several factors can cause an inflammatory response, including reperfusion of significant organs after ischemia, endotoxin released from the inflamed gut, and mechanical surgical trauma (47). Furthermore, some studies found that global injury from CPB and cardiac arrest and localized injury from non-CPB can cause cardiac ischemia-reperfusion injury, while myocardial reperfusion injury activates neutrophils, triggering an inflammatory response (48).

### Inflammatory response during myocardial ischemia and reperfusion

Ischemia and reperfusion are pathological conditions whereby the recanalization and reoxygenation of blood flow are usually associated with increased tissue damage and inflammatory response, called reperfusion injury (49). The earliest feature of cardiomyocyte ischemia is the depletion of intracellular ATP. The reduction of molecular oxygen inhibits the coupling between the respiratory chain and oxidative phosphorylation and inhibits ATP synthesis. Therefore, cellular ischemia and hypoxia disrupt the balance between energy production and utilization, and roughly 95% of the energy produced by mitochondrial oxidative metabolism is stored in the ATP molecule, which is essential for the heart’s metabolic activities and mechanical functions. During cellular ischemia, mitochondrial energy decreases production and is accompanied by abnormal accumulation and depletion of several intracellular metabolites, including a rise in intracellular calcium ions and lactate and a decline in ATP levels. Ample evidence substantiates that ATP depletion increases cardiomyocyte membrane permeability and intracellular calcium ion concentration and drives the activation of calcium-dependent phospholipase and proteolytic enzymes, which may affect endothelial production, cell oxygen radical production and leukocyte-endothelial interactions (50–52). Myocardial reperfusion injury leads to inflammation and oxidative stress and produces various inflammatory cytokines and reactive oxygen species (ROS), inhibiting cardiac function and apoptosis (53). Many experimental models found that the myocardium produces IL-6 during ischemia-reperfusion injury (54, 55). Besides IL-6, some inflammatory factors can be generated to a certain extent in the heart. For instance, IL-8 release during myocardial ischemia can stimulate the upregulation of adhesion molecules on different cell types (56). Besides the above pro-inflammatory factors, cardiomyocytes produce IL-18 and IL-1 pro-inflammatory cytokines (57–59). In addition, heart cells produce anti-inflammatory factors like IL-10 (60). Hence, cardiomyocytes are a source of inflammatory factors and markers, especially during ischemia-reperfusion injury.

## Inflammation and the pathogenesis of postoperative delirium

### Effects of different inflammatory factors on cardiac performance

It has been established that cytokines, including pro- and anti-inflammatory factors, can either damage or protect the myocardium during inflammation. Moreover, these effects can be achieved by acting directly on cardiomyocytes or altering the levels of cardiac injury markers. IL-6 production has been associated with adverse inotropic effects, myocardial stunning, and reduced neutrophil infiltration (61–63). It has been suggested that myocardial depression is associated with increased nitric oxide (NO) production, increasing intracellular cyclic guanosine monophosphate and activating cyclic guanosine monophosphate-dependent protein kinase to inhibit L-type Ca^2+^ channels inducing adverse inotropic effects (64, 65). In addition to IL-6, IL-8 locally generated in the heart exacerbates cardiac injury by enhancing leukocyte activation and aggregation. Another cytokine, IL-18, can reportedly promote the activation of pro-apoptotic signaling pathways and induce endothelial cell death (66). Besides the pro-inflammatory markers described above, anti-inflammatory markers also influence cardiac function. For instance, IL-10 may attenuate reperfusion injury via inhibiting neutrophil infiltration into the myocardium (60). Furthermore, increased inflammatory markers, chemokines, and some inflammatory markers are often accompanied by endothelial dysfunction and blood-brain barrier (BBB) disruption (67). Neuroinflammation can cause neuron damage in the brain and concomitant microglial activation leading to delirium (68).

### Inflammatory response and factors that affect the occurrence of postoperative delirium

Although the exact mechanisms of delirium onset remain unclear, there are multiple hypotheses regarding its pathophysiological mechanisms, of which neuroinflammation has attracted the most attention. During cardiac surgery, stress factors such as CPB, surgical trauma and ischemia-reperfusion injury lead to significant inflammation, which also provides a pathway for neuroinflammation processes. POD has long been thought to respond to physiological disorders caused by CPB management, explaining the term “pump-head” (69–71). However, recent studies revealed similar POD incidence and inflammatory marker levels, irrespective of CPB use during the surgical procedure (71–75). Hence, it has been demonstrated that avoiding CPB does not improve cognitive functions. CPB itself can trigger SIRS, which contributes to BBB leakage and the development of neuroinflammation (73, 76, 77), as well as also can activate pro-inflammatory factors produced by macrophages and monocytes to increase BBB permeability and change neurotransmission, which may play a key role in causing POD (78). To verify whether SIRS is involved in POD, the optimal steroid type dose and administration time, i.e., dexamethasone, 0.1 mg/kg, 10 h before surgery, have been determined (79). Indeed, inflammation may play a crucial role in long-term cognitive function. However, there is still an urgent need for more research in this area to determine its exact role.

## Oxidative stress during cardiac surgery

Redox signaling is involved in changes in ROS levels and various processes, such as stress response pathways, homeostasis and cardiac remodeling and fibrosis (80–83). ROS are a natural by-product of the normal oxygen metabolism process produced in mitochondria where aerobic metabolism occurs. It is well-recognized that cells use various defense mechanisms to protect themselves from ROS. However, ROS levels increase more dramatically during stress or inflammation than endogenous antioxidant capacity, leading to oxidative stress, thereby causing substantial damage to many cellular molecules (80, 81, 84). Preoperative coronary atherosclerosis, diabetes mellitus, and related kidney and lung diseases have been associated with oxidative stress, which can be severe during CPB (40).

### Of reactive oxygen species during surgery

Mounting evidence substantiates that during cardiac surgery, the body produces large amounts of unstable free radicals (85). Under physiological conditions, ROS and nitrogen can act as messengers for normal cellular functions. However, under oxidative stress conditions, they can disrupt intracellular Ca^2+^ homeostasis and lead to cell death (86). Different coagulation and pro-inflammation pathways, activation of the survival cascade and altered redox status are related to CPB (87–89). Although significant improvements have been made over the years in cardiac surgery, oxidative stress and inflammation remain significant issues to be addressed in the CPB period (90, 91). As described above, during CPB, hemolysis, ischemia-reperfusion injury, and neutrophil activation exert a crucial effect on the activation of oxidative stress and associated pro-inflammatory and pro-apoptotic signaling pathways, affecting multiple organs, including the cardiac myocardium, lung, and kidney, as well as influencing clinical outcomes. Importantly, the primary source of ROS in cardiac surgery under CPB is neutrophils (92), which are activated by cytokines from the systemic circulation, coronary vessels, and cardiomyocytes. For example, cytokines can stimulate the upregulation of adhesion molecules on cardiomyocytes, causing neutrophils to adhere and release ROS and proteolytic enzymes (56).

**FIGURE 2:**
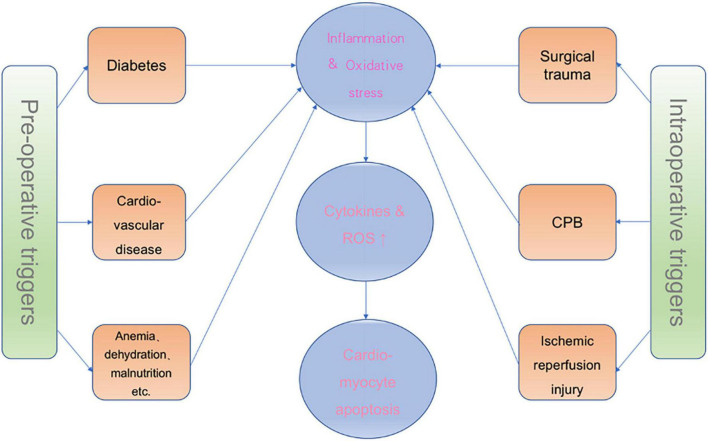
Effects of CPB and ischemia-reperfusion injury on POD and the source of ROS. NAPDH II, NADPH oxidase 2; HK II, hexokinase II; MAPK, mitogen-activated protein kinase; NF-kB, nuclear transcription factor-κB; TNF-α:tumor necrosis factor-α; BBB, blood-brain barrier; MPTP, mitochondrial permeability transition pore.

In contrast, the myocardium produces ROS during ischemia-reperfusion injury (53), mainly through activation of NADPH oxidase 2, and increases mitochondrial ROS through metabolic overload and reduced hexokinase II binding on mitochondria (93). In addition, ROS can promote nitrosylation, carbonylation, disulfide bond formation and glutathionylation to regulate the activity of signaling proteins, thereby inducing the activation of pro-inflammatory and pro-apoptotic signaling pathways, such as MAPK and NF-κB signaling pathways, and leading to cytoskeleton disruption and cell tube damage (94–97) (Figure 2).

### Reactive oxygen species mediate the pathogenesis of postoperative delirium

During surgery, especially during CPB and ischemia-reperfusion injury, the levels of ROS are markedly increased while the antioxidant capacity is decreased (98). Recent studies have shown that decreased antioxidant capacity is an essential feature of conditions such as major depression and cognitive impairment. Besides, it has been documented that patients with cerebrovascular or psychiatric diseases have low plasma antioxidant capacity (99, 100), and oxidative stress mediates neuronal damage (101, 102).

## Perioperative risk factors

Many risk factors are associated with postoperative delirium. While some may predispose patients to delirium, other risk factors precipitate delirium such as medications given during the perioperative period. Predisposing factors must be considered when performing treatment in the clinical setting, especially in patients already at high risk for postoperative delirium. For controllable risk factors, optimization should be performed in the perioperative period. Based on evidence and consensus statements of the European Society of Anesthesiology guidelines (103) and other studies, we propose some perioperative risk factors that may contribute to postoperative delirium and are listed in Table 2.

**TABLE 2:** Perioperative risk factors for delirium after cardiac surgery.

Preoperative risk factors	Intra-postoperative risk factors
Age ≥ 60 years	CPB time
Preoperative co-morbidities (cerebrovascular diseases such as stroke, carotid stenosis, TIA; atrial fibrillation, diabetes, anemia, depression, Parkinson’s, dementia)	Type of surgery Intraoperative bleeding (transfusion of red blood cells and platelets)
Frailty (malnutrition, hypoalbuminemia, hypercholesterolemia, high levels of inflammation, muscular atrophy, etc.)	Hypothermia
Prolonged preoperative fasting and dehydration	Emergency surgery
Hearing impairment and visual impairment	Depth of anesthesia and medications
Chronic alcohol/substance abuse	Pain

TIA, Transient ischemic attack, CPB, Cardiopulmonary bypass.

### Preoperative risk factors

A great number of current studies have shown that advanced age is a recognized independent risk factor for postoperative delirium and that the risk of POD increases progressively with age (13, 104). In a meta-analysis by Chen et al. (105), preoperative carotid stenosis, diabetes mellitus, hypertension, left ventricular ejection fraction percentage, preoperative cognitive impairment, and NYHA class III or IV were found to be risk factors for postoperative cardiac delirium. Among them, a study by Junichiro Miyazaki et al. found that carotid plaque > 50% was a significant predictor of postoperative stroke or transient cerebral ischemia (TIA) or delirium in cardiac surgery (non-extracorporeal CABG). In addition to this, a study by Trabold et al. (13) found that preoperative anemia, dehydration, electrolyte abnormalities and signs of malnutrition were also risk factors for postoperative delirium. However, it remains to be elucidated whether intervention of these risk factors before surgery affects the outcome of delirium, and therefore, a large number of studies should be needed to investigate whether preoperative prevention and optimization of these risk factors has an impact on the incidence of delirium after cardiac surgery.

### Intra- and postoperative risk factors

Whether the duration of CPB contributes to delirium after cardiac surgery and yielded conflicting results. Several studies have found an independent association between CPB duration and POD (106, 107), such as in a study by O’neal et al. (107), which found that CPB duration was associated with a significantly increased risk of delirium in patients who underwent CABG. Whereas in Smith et al. (108) retrospectively examined 12 studies related to this issue and found that six studies resulted in postoperative delirium; five found no effect and one study found a negative association. The duration of CPB depends on the complexity of the procedure, procedures with higher complexity have an increased incidence of postoperative delirium. The incidence of delirium is higher in valve surgery and CABG compared to valve replacement or repair, and higher in more complex procedures such as replacement of multiple valves, aortic reconstruction, and deep hypothermic extracorporeal circulation. Whether valve surgery itself causes an increased rate of cerebral embolism leading to a higher incidence of POD or whether the length of surgery or CPB causes POD is unknown and therefore requires more research to explore and confirm. In addition, Whether the presence or absence of extracorporeal circulation in coronary artery grafting affects the incidence of postoperative delirium has been a question that many scholars have tried to investigate and explain. It is well known that during the period of CPB, the direct exposure of blood to the foreign body surface activates immune mediators, as well as CPB-induced leukocyte/platelet activation, procoagulation and fibrinolysis mediated by thrombin/fibrin, leading to elevated proinflammatory mediators and activation of systemic inflammatory response, thus CABG surgery triggers a complex pro-thrombotic and pro-inflammatory response which is thought to damage the BB, leading to neuroinflammation and consequent neurological dysfunction. The off-pump CABG procedure, on the other hand, is thought to trigger a lesser inflammatory response than CABG due to the absence of CPB. The current study reports conflicting results for the two surgical modalities on POD, with Gaudino’s (109) study finding no significant difference in the incidence of postoperative delirium between CABG under non-extracorporeal and extracorporeal circulation. In contrast, in a study by O’neal et al. (107), the duration of CPB was found to be associated with a significantly increased risk of delirium in patients receiving CABG. Therefore, more clinical experimental studies are needed to explore and confirm this. Intraoperative transfusion of hemoglobin and platelets can also affect the postoperative morbidity. It is well known that the input of processed and stored blood products can cause severe systemic inflammation. Studies have found (110), that perioperative blood transfusion is an independent risk factor for postoperative delirium. In addition to this, several studies (111, 112), have shown that there is an independent risk factor for POD during perioperative allogeneic transfusion and that there is a dose-dependent relationship between the amount of blood transfused and the risk of POD.

Due to the specificity of cardiac surgery, the requirements of hypothermia need to be met during the procedure, and prolonged and sustained hypothermia can induce burst suppression of the EEG. In addition, the depth of anesthesia gradually increases as the temperature decreases, and perioperative neurodetectors commonly used in cardiac surgery, such as cerebral oxygen saturation or EEG detectors (i.e., dual-frequency index BIS), have been shown to help predict postoperative delirium. In recent years, many studies (113–115) have demonstrated the effect of anesthetic depth monitoring on neurological complications such as POD, POCD, and cerebrovascular accidents. A study by Perez-Otal (116) found that obtaining adequate anesthetic depth through intraoperative neuromonitoring and adjusting the anesthetic dose to avoid overuse of anesthetic drugs helped improve postoperative cognition in the elderly. Cardiac surgery is commonly associated with postoperative pain due to large surgical invasions and surgical indwelling tubes. Pain causes an acute stress response and an immediate cognitive burden (117) and increases the risk of other postoperative complications, such as pulmonary atelectasis, which may also contribute to POD. A study by Subramaniam (118) found that postoperative administration of analgesic drugs combined with sedative drugs significantly reduced the incidence of in-hospital delirium in elderly patients undergoing cardiac surgery.

For cardiac surgery, perioperative neurological dysfunction caused by brain injury, in addition to delirium, stroke is a topic that has generated much debate among scholars. The three major causes of neurologic dysfunction and injury during cardiac surgery are microemboli, hypoperfusion, and a generalized inflammatory reaction. Many intraoperative strokes are the result of the embolization of atherosclerotic material from the aorta and brachiocephalic vessels. In addition to this, air and fat emboli blocking blood vessels can also cause neuronal damage. The important principles to reduce emboli are anticoagulation, filtration of blood, removal of air and avoidance of atherosclerotic emboli. Many intraoperative monitoring modalities are available to reduce the occurrence of such risks, such as TEE to help surgeons determine the optimal aortic cross-clamping and cannulation position, which can be effective in avoiding potential embolization of atherosclerotic plaques, and intravenous reservoirs and arterial line filters in CPB circuits to effectively eliminate air and fat emboli. Various brain protection techniques are also used during cardiac surgery, including hypothermia, cerebral perfusion modalities, and pharmacological protection. Maintaining optimal cerebral perfusion by maintaining MAP at a perfusion pressure within the autonomic range of the brain during surgery may also be an important component in reducing brain injury. In addition to this, lowering brain temperature may reduce cerebral blood flow and excitotoxicity to protect against ischemic neuronal injury. There are no clear guidelines for the prevention of intraoperative stroke, but some intraoperative monitoring strategies may help to avoid stroke. In general, non-pharmacological strategies include monitoring of brain oxygenation and perfusion with devices such as near infrared spectroscopy and Transcranial Doppler help. Epiaortic and transesophageal echocardiography visualize aorta pathology, enabling the surgeon to sidestep atheromatous segments. Additionally can the use of specially designed aortic cannulae and filters help to reduce embolization. Brain perfusion can be improved by using antero- or retrograde cerebral perfusion during deep hypothermic circulatory arrest, by tightly monitoring mean arterial blood pressure and hemodilution. Controlling perioperative temperature and glucose levels may additionally help to ameliorate secondary damage. For perioperative neurological dysfunction caused by brain injury after cardiac surgery, current studies suggest that microemboli, hypoperfusion and systemic inflammatory response are the results. Among them, embolic occlusion and cerebral hypoperfusion are considered to be important causes of intraoperative stroke. Although the risk factors for causing POD are currently identified as being very similar to those for stroke, efforts to link the extent of for emboli and POD have been inconsistent.

## Preoperative interventions and management

### The function of related anti-inflammatory agents and antioxidants in reducing postoperative delirium

#### Parecoxib

Parecoxib is a non-steroidal anti-inflammation drug that can selectively block the effect of cyclooxygenase-2 (COX-2) and can be rapidly converted to the active metabolite valdecoxib in the body. It is widely used clinically in treating osteoarthritis, rheumatoid arthritis, and postoperative pain relief (119, 120). A recent study (121) showed that parecoxib has neuroprotective effects. Some studies have found that treatment of primary cultured rat astrocytes with H_2_O_2_, a strong oxidizing agent, led to oxidative damage and was used to establish an oxidative stress model. These results substantiated that parecoxib exerts a protective effect against H_2_O_2_-induced oxidative damage in rat astrocytes, and its mechanism of action may be related to a reduction in cellular ROS levels, a decrease of apoptosis rate, inhibition of aquaporin-4 (AQP4) expression, a decrease of b-cell lymphoma-2 (Bcl-2) associated X protein (Bax) expression, and increase of BCL-2 and brain-derived neurotrophic factor (BDNF) expression.

#### Statins

Statins are HMG-CoA reductase inhibitors with pleiotropic effects, including anti-inflammatory, antioxidant, immunomodulatory, and antithrombotic properties (122–124). It is well-established that statins modulate the expression of pro-inflammatory factors such as IL-8, IL-6 and monocyte chemoattractant protein-1 (MCP-1) levels, thereby exerting anti-inflammatory and cardioprotective effects during cardiac surgery (125). Intriguingly, statins can modulate NAPDH oxidase enzyme activity, thus attenuating ROS production and exerting an antioxidant effect (126). An observational study by Katznelson et al. (127) found that preoperative administration of statins was associated with a reduced risk of POD by analyzing 1059 patients undergoing cardiac surgery with CPB, and the protective effect on POD was more pronounced in patients ≥60 years of age. However, some studies (128, 129) found no association between preoperative statin administration and POD after CPB by analyzing IL-1, T-α and C-reactive protein levels in patients undergoing coronary artery bypass grafting (CABG) surgery. To account for this controversial effect of statins on POD, some researchers proposed multifactorial and complex pathogenic mechanisms that cause POD (e.g., embolic events occurring in CPB, cardiac output and hypoperfusion and hypoxic events, and prolonged postoperative ICU stay can inhibit the anti-inflammatory effects of statins) (8, 129–132). Therefore, the protective effect of statins on POD in cardiac surgery has not been clearly defined, emphasizing the need for more studies.

#### Melatonin

Melatonin is a natural hormone produced by the pineal gland in the brain, synthesized from tryptophan and has been established to participate in sleep-wake cycle regulation (133). Melatonin production reportedly quickly declines with age and can exert antioxidant effects by scavenging free radicals, stimulating endogenous antioxidant enzymes, improving the efficiency of other antioxidants, and protecting mitochondria from oxidative stress by affecting mitochondrial membrane potential (134, 135). In addition, a growing body of literature suggests that melatonin exerts anti-inflammatory effects in acute or chronic inflammatory processes (136). In the study by El-Shenawy and Carrasco (137, 138), it was found that in rats pretreated with melatonin, the inflammatory response and the levels of pro-inflammatory factors, such as IL-1β and TNF-α, were decreased, while the levels of anti-inflammatory factor IL-4 were increased. The above factors suggest a potential role of melatonin in POD (139). A meta-analysis by Asleson et al. (140) suggested an association between melatonin and POD prevention. Although contradictory findings have been reported in the literature, the lack of validity may be due to sampling size effects. Therefore, larger sample sizes and more complex randomized controlled trials are needed to determine the efficacy of melatonin on POD.

#### Dexmedetomidine

Dexmedetomidine (DEX) is a potent α2-adrenoceptor agonist and a common and vital adjunct during general anesthesia. In addition to a low hemodynamic impact, it can reduce the dose of intraoperative anesthetic and analgesic drugs and improve patient comfort postoperatively (141). Recent studies have demonstrated that DEX can exert anti-inflammatory effects by reducing the expression of various pro-inflammatory factors while protecting organ tissues from injury (142, 143). In addition, DEX may alleviate central nervous system (CNS) damage through antioxidant effects (144). Animal experiments by Wu and Hu et al. (145, 146) have reported that DEX provides neuroprotective effects and improves cognitive function after cardiac surgery. Besides, a meta-analysis of 26 randomized controlled trials by Wan et al. found that peri-operative DEX treatment in patients undergoing general anesthesia could significantly reduce the incidence of POD and inflammation compared with controls (147). Overall, DEX exhibits therapeutic effects against POD and inflammation.

## Conclusion

Postoperative delirium (POD) is a common complication after cardiac surgery and can affect patient survival and mortality. Although many risk factors have been established regarding POD, the molecular mechanisms of the development of delirium are not fully understood. Current evidence suggests that inflammation caused by preoperative disease status, CPB, and ischemia-reperfusion injury may lead to delirium. Indeed, more research and randomized experiments in this field are warranted to confirm and provide new ideas for the clinical treatment of POD. Although the precise cellular mechanisms and pathways for POD occurrence are still difficult to resolve. Some perioperative interventions and pharmacological prophylaxis have shown promising results for the improvement of delirium. However, more work needs to be done before they can be routinely incorporated into clinical practice. In particular, a better understanding of the role in the development of POD is an important step toward developing more effective treatments and optimizing the conditions for anti-inflammatory and antioxidant-based therapeutic interventions. Eventually, this review also has some limitations. There is a large body of literature describing the possible relevance of inflammation and oxidative stress to POD, but in some studies the relationship between these factors was found to be inconsistent. However, some perioperative interventions and pharmacological prophylaxis have shown good results in improving delirium, and it is certain that POD has a complex association with inflammation and oxidative stress. Therefore, more work is needed before they can be routinely incorporated into clinical practice, especially in the development of more effective therapeutic approaches and in the optimization of conditions for anti-inflammatory and antioxidant-based therapeutic interventions.

## Author contributions

YP helped described this review and wrote the manuscript. YL helped with the literature search and revising the manuscript. YZ helped with the literature search. HW, JL, LH, HL, XX, LG, and XW helped revise the manuscript. All authors contributed to the article and approved the submitted version.

## References

[1] Mattison MLP. Delirium. *Ann Intern Med.* (2020) 173:Itc49–64. 10.7326/AITC202010060 33017552

[2] Young J Inouye SK. Delirium in older people. *BMJ.* (2007) 334:842–6. 10.1136/bmj.39169.706574.AD 17446616 PMC1853193

[3] Deiner S Silverstein JH. Postoperative delirium and cognitive dysfunction. *Br J Anaesth.* (2009) 103(Suppl 1):i41–6. 10.1093/bja/aep291 20007989 PMC2791855

[4] Mittal V Muralee S Williamson D McEnerney N Thomas J Cash M Review: delirium in the elderly: a comprehensive review. *Am J Alzheimers Dis Other Demen.* (2011) 26:97–109. 10.1177/1533317510397331 21285047 PMC10845585

[5] Miller RR III Ely EW. Delirium and cognitive dysfunction in the intensive care unit. *Semin Respir Crit Care Med.* (2006) 27:210–20. 10.1055/s-2006-945532 16791755

[6] Sockalingam S Parekh N Bogoch II Sun J Mahtani R Beach C Delirium in the postoperative cardiac patient: a review. *J Card Surg.* (2005) 20:560–7. 10.1111/j.1540-8191.2005.00134.x 16309412

[7] Lee SJ Jung SH Lee SU Lim JY Yoon KS Lee SY. Postoperative delirium after hip surgery is a potential risk factor for incident dementia: a systematic review and meta-analysis of prospective studies. *Arch Gerontol Geriatr.* (2020) 87:103977. 10.1016/j.archger.2019.103977 31751902

[8] Kazmierski J Kowman M Banach M Fendler W Okonski P Banys A Incidence and predictors of delirium after cardiac surgery: results from The IPDACS Study. *J Psychosom Res.* (2010) 69:179–85. 10.1016/j.jpsychores.2010.02.009 20624517

[9] Djaiani G Silverton N Fedorko L Carroll J Styra R Rao V Dexmedetomidine versus propofol sedation reduces delirium after cardiac surgery: a randomized controlled trial. *Anesthesiology.* (2016) 124:362–8. 10.1097/ALN.0000000000000951 26575144

[10] Figueiredo-Pereira ME Rockwell P Schmidt-Glenewinkel T Serrano P. Neuroinflammation and J2 prostaglandins: linking impairment of the ubiquitin-proteasome pathway and mitochondria to neurodegeneration. *Front Mol Neurosci.* (2014) 7:104. 10.3389/fnmol.2014.00104 25628533 PMC4292445

[11] Ruitenberg A Ott A van Swieten JC Hofman A Breteler MM. Incidence of dementia: does gender make a difference? *Neurobiol Aging.* (2001) 22:575–80. 10.1016/S0197-4580(01)00231-7 11445258

[12] Tse L Schwarz SK Bowering JB Moore RL Burns KD Richford CM Pharmacological risk factors for delirium after cardiac surgery: a review. *Curr Neuropharmacol.* (2012) 10:181–96. 10.2174/157015912803217332 23449337 PMC3468873

[13] Trabold B Metterlein T. Postoperative delirium: risk factors, prevention, and treatment. *J Cardiothorac Vasc Anesth.* (2014) 28:1352–60. 10.1053/j.jvca.2014.03.017 25281048

[14] Laffey JG Boylan JF Cheng DC. The systemic inflammatory response to cardiac surgery: implications for the anesthesiologist. *Anesthesiology.* (2002) 97:215–52. 10.1097/00000542-200207000-00030 12131125

[15] Clermont G Vergely C Jazayeri S Lahet JJ Goudeau JJ Lecour S Systemic free radical activation is a major event involved in myocardial oxidative stress related to cardiopulmonary bypass. *Anesthesiology.* (2002) 96:80–7. 10.1097/00000542-200201000-00019 11753006

[16] Hshieh TT Fong TG Marcantonio ER Inouye SK. Cholinergic deficiency hypothesis in delirium: a synthesis of current evidence. *J Gerontol A Biol Sci Med Sci.* (2008) 63:764–72. 10.1093/gerona/63.7.764 18693233 PMC2917793

[17] Maldonado JR. Pathoetiological model of delirium: a comprehensive understanding of the neurobiology of delirium and an evidence-based approach to prevention and treatment. *Crit Care Clin.* (2008) 24:789–856. 10.1016/j.ccc.2008.06.004 18929943

[18] Whitlock EL Vannucci A Avidan MS. Postoperative delirium. *Minerva Anestesiol.* (2011) 77:448–56. 21483389 PMC3615670

[19] Li J Meng D Chang C Fu B Xie C Wu Z Risk factors for delirium after coronary artery bypass grafting in elderly patients. *Ann Transl Med.* (2021) 9:1666. 10.21037/atm-21-5160 34988175 PMC8667104

[20] Lechowicz K Szylińska A Listewnik M Drożdżal S Tomska N Rotter I Cardiac delirium index for predicting the occurrence of postoperative delirium in adult patients after coronary artery bypass grafting. *Clin Interv Aging.* (2021) 16:487–95. 10.2147/CIA.S302526 33762820 PMC7982438

[21] Brown CHT Laflam A Max L Lymar D Neufeld KJ Tian J The impact of delirium after cardiac surgical procedures on postoperative resource use. *Ann Thorac Surg.* (2016) 101:1663–9. 10.1016/j.athoracsur.2015.12.074 27041454 PMC5406132

[22] Norkiene I Ringaitiene D Misiuriene I Samalavicius R Bubulis R Baublys A Incidence and precipitating factors of delirium after coronary artery bypass grafting. *Scand Cardiovasc J.* (2007) 41:180–5. 10.1080/14017430701302490 17487768

[23] Liu Z Pang X Zhang X Cao G Fang C Wu S. Incidence and risk factors of delirium in patients after type-A aortic dissection surgery. *J Cardiothorac Vasc Anesth.* (2017) 31:1996–9. 10.1053/j.jvca.2016.11.011 28109683

[24] Cai S Zhang X Pan W Latour JM Zheng J Zhong J Prevalence, predictors, and early outcomes of post-operative delirium in patients with type A aortic dissection during intensive care unit stay. *Front Med.* (2020) 7:572581. 10.3389/fmed.2020.572581 33072785 PMC7544982

[25] Liu J Yang F Luo S Li C Liu W Liu Y Incidence, predictors and outcomes of delirium in complicated type B aortic dissection patients after thoracic endovascular aortic repair. *Clin Interv Aging.* (2021) 16:1581–9. 10.2147/CIA.S328657 34471348 PMC8405167

[26] He J Ling Q Chen Y. Construction and application of a model for predicting the risk of delirium in postoperative patients with type a aortic dissection. *Front Surg.* (2021) 8:772675. 10.3389/fsurg.2021.772675 34869569 PMC8636852

[27] Humbert M Büla CJ Muller O Krief H Monney P. Delirium in older patients undergoing aortic valve replacement: incidence, predictors, and cognitive prognosis. *BMC Geriatr.* (2021) 21:153. 10.1186/s12877-021-02100-5 33653285 PMC7927377

[28] Rao A Shi SM Afilalo J Popma JJ Khabbaz KR Laham RJ Physical performance and risk of postoperative delirium in older adults undergoing aortic valve replacement. *Clin Interv Aging.* (2020) 15:1471–9. 10.2147/CIA.S257079 32921993 PMC7455771

[29] Shi SM Sung M Afilalo J Lipsitz LA Kim CA Popma JJ Delirium incidence and functional outcomes after transcatheter and surgical aortic valve replacement. *J Am Geriatr Soc.* (2019) 67:1393–401. 10.1111/jgs.15867 30882905 PMC6612597

[30] Wesselink EM Abawi M Kooistra NHM Kappen TH Agostoni P Emmelot-Vonk M. Intraoperative hypotension and delirium among older adults undergoing transcatheter aortic valve replacement. *J Am Geriatr Soc.* (2021) 69:3177–85. 10.1111/jgs.17361 34612514 PMC9293424

[31] van der Wulp K van Wely M van Heijningen L van Bakel B Schoon Y Verkroost M. Delirium after transcatheter aortic valve implantation under general anesthesia: incidence, predictors, and relation to long-term survival. *J Am Geriatr Soc.* (2019) 67:2325–30. 10.1111/jgs.16087 31342524 PMC6899857

[32] Goudzwaard JA de Ronde-Tillmans M de Jager TAJ Lenzen MJ Nuis RJ van Mieghem NM. Incidence, determinants and consequences of delirium in older patients after transcatheter aortic valve implantation. *Age Ageing.* (2020) 49:389–94. 10.1093/ageing/afaa001 32091096 PMC7577406

[33] Körber MI Schäfer M Vimalathasan R Mauri V Iliadis C Metze C. Prevalence and impact of post-procedural delirium after percutaneous repair of mitral and tricuspid valves. *JACC Cardiovasc Interv.* (2021) 14:588–90. 10.1016/j.jcin.2020.11.031 33663792

[34] Wiedemann D Bernhard D Laufer G Kocher A. The elderly patient and cardiac surgery – a mini-review. *Gerontology.* (2010) 56:241–9. 10.1159/000248761 19828936

[35] Pol RA van Leeuwen BL Reijnen MM Zeebregts CJ. The relation between atherosclerosis and the occurrence of postoperative delirium in vascular surgery patients. *Vasc Med.* (2012) 17:116–22. 10.1177/1358863X11429723 22302037

[36] Zakkar M Guida G Suleiman MS Angelini GD. Cardiopulmonary bypass and oxidative stress. *Oxid Med Cell Longev.* (2015) 2015:189863. 10.1155/2015/189863 25722792 PMC4334937

[37] Kobiyama K Ley K. Atherosclerosis. *Circ Res.* (2018) 123:1118–20. 10.1161/CIRCRESAHA.118.313816 30359201 PMC6298754

[38] Libby P Crea F. Clinical implications of inflammation for cardiovascular primary prevention. *Eur Heart J.* (2010) 31:777–83. 10.1093/eurheartj/ehq022 20185554

[39] Libby P. Inflammation in atherosclerosis. *Arterioscler Thromb Vasc Biol.* (2012) 32:2045–51. 10.1161/ATVBAHA.108.179705 22895665 PMC3422754

[40] Pearson TA Mensah GA Alexander RW Anderson JL Cannon RO III Criqui M Markers of inflammation and cardiovascular disease: application to clinical and public health practice: a statement for healthcare professionals from the Centers for Disease Control and Prevention and the American Heart Association. *Circulation.* (2003) 107:499–511. 10.1161/01.CIR.0000052939.59093.45 12551878

[41] Grundy SM Bazzarre T Cleeman J D’Agostino RB Sr Hill M Houston-Miller N. Prevention conference V: Beyond secondary prevention: identifying the high-risk patient for primary prevention: medical office assessment: Writing Group. *Circulation.* (2000) 101:E3–11. 10.1161/01.CIR.101.1.e3 10618316

[42] Alam A Hana Z Jin Z Suen KC Ma D. Surgery, neuroinflammation and cognitive impairment. *EBioMedicine.* (2018) 37:547–56. 10.1016/j.ebiom.2018.10.021 30348620 PMC6284418

[43] Wan S LeClerc JL Vincent JL. Inflammatory response to cardiopulmonary bypass: mechanisms involved and possible therapeutic strategies. *Chest.* (1997) 112:676–92. 10.1378/chest.112.3.676 9315800

[44] Wu Q Purusram G Wang H Yuan R Xie W Gui P The efficacy of parecoxib on systemic inflammatory response associated with cardiopulmonary bypass during cardiac surgery. *Br J Clin Pharmacol.* (2013) 75:769–78. 10.1111/j.1365-2125.2012.04393.x 22835079 PMC3575943

[45] Pintar T Collard CD. The systemic inflammatory response to cardiopulmonary bypass. *Anesthesiol Clin North Am.* (2003) 21:453–64. 10.1016/S0889-8537(03)00039-7 14562560

[46] Okubo N Hatori N Ochi M Tanaka S. Comparison of m-RNA expression for inflammatory mediators in leukocytes between on-pump and off-pump coronary artery bypass grafting. *Ann Thorac Cardiovasc Surg.* (2003) 9:43–9. 12667129

[47] Asimakopoulos G. Mechanisms of the systemic inflammatory response. *Perfusion.* (1999) 14:269–77. 10.1177/026765919901400406 10456781

[48] Petzelbauer P Zacharowski PA Miyazaki Y Friedl P Wickenhauser G Castellino FJ The fibrin-derived peptide Bbeta15-42 protects the myocardium against ischemia-reperfusion injury. *Nat Med.* (2005) 11:298–304. 10.1038/nm1198 15723073

[49] Yellon DM Hausenloy DJ. Myocardial reperfusion injury. *N Engl J Med.* (2007) 357:1121–35. 10.1056/NEJMra071667 17855673

[50] Chien KR Han A Sen A Buja LM Willerson JT. Accumulation of unesterified arachidonic acid in ischemic canine myocardium. relationship to a phosphatidylcholine deacylation-reacylation cycle and the depletion of membrane phospholipids. *Circ Res.* (1984) 54:313–22. 10.1161/01.RES.54.3.313 6421507

[51] Suzuki M Inauen W Kvietys PR Grisham MB Meininger C Schelling ME Superoxide mediates reperfusion-induced leukocyte-endothelial cell interactions. *Am J Physiol.* (1989) 257:H1740–5. 10.1152/ajpheart.1989.257.5.H1740 2556051

[52] Valen G Vaage J. Toxic oxygen metabolites and leukocytes in reperfusion injury. a review. *Scand J Thorac Cardiovasc Surg Suppl.* (1993) 41:19–29. 10.3109/14017439309100155 8184290

[53] Wang M Baker L Tsai BM Meldrum KK Meldrum DR. Sex differences in the myocardial inflammatory response to ischemia-reperfusion injury. *Am J Physiol Endocrinol Metab.* (2005) 288:E321–6. 10.1152/ajpendo.00278.2004 15367393

[54] Sawa Y Ichikawa H Kagisaki K Ohata T Matsuda H. Interleukin-6 derived from hypoxic myocytes promotes neutrophil-mediated reperfusion injury in myocardium. *J Thorac Cardiovasc Surg.* (1998) 116:511–7. 10.1016/S0022-5223(98)70018-2 9731794

[55] Chandrasekar B Mitchell DH Colston JT Freeman GL. Regulation of CCAAT/Enhancer binding protein, interleukin-6, interleukin-6 receptor, and gp130 expression during myocardial ischemia/reperfusion. *Circulation.* (1999) 99:427–33. 10.1161/01.CIR.99.3.427 9918531

[56] Ren G Dewald O Frangogiannis NG. Inflammatory mechanisms in myocardial infarction. *Curr Drug Targets Inflamm Allergy.* (2003) 2:242–56. 10.2174/1568010033484098 14561159

[57] Deten A Volz HC Holzl A Briest W Zimmer HG. Effect of propranolol on cardiac cytokine expression after myocardial infarction in rats. *Mol Cell Biochem.* (2003) 251:127–37. 10.1007/978-1-4419-9238-3_18 14575314

[58] Matsumori A Igata H Ono K Iwasaki A Miyamoto T Nishio R High doses of digitalis increase the myocardial production of proinflammatory cytokines and worsen myocardial injury in viral myocarditis: a possible mechanism of digitalis toxicity. *Jpn Circ J.* (1999) 63:934–40. 10.1253/jcj.63.934 10614837

[59] Pomerantz BJ Reznikov LL Harken AH Dinarello CA. Inhibition of caspase 1 reduces human myocardial ischemic dysfunction via inhibition of IL-18 and IL-1beta. *Proc Natl Acad Sci U.S.A.* (2001) 98:2871–6. 10.1073/pnas.041611398 11226333 PMC30232

[60] Jones SP Trocha SD Lefer DJ. Cardioprotective actions of endogenous IL-10 are independent of iNOS. *Am J Physiol Heart Circ Physiol.* (2001) 281:H48–52. 10.1152/ajpheart.2001.281.1.H48 11406467

[61] Finkel MS Oddis CV Jacob TD Watkins SC Hattler BG Simmons RL. Negative inotropic effects of cytokines on the heart mediated by nitric oxide. *Science.* (1992) 257:387–9. 10.1126/science.1631560 1631560

[62] Zahler S Massoudy P Hartl H Hähnel C Meisner H Becker BF. Acute cardiac inflammatory responses to postischemic reperfusion during cardiopulmonary bypass. *Cardiovasc Res.* (1999) 41:722–30. 10.1016/S0008-6363(98)00229-6 10435044

[63] Cuzzocrea S De Sarro G Costantino G Ciliberto G Mazzon E De Sarro A. IL-6 knock-out mice exhibit resistance to splanchnic artery occlusion shock. *J Leukoc Biol.* (1999) 66:471–80. 10.1002/jlb.66.3.471 10496318

[64] Stangl V Baumann G Stangl K Felix SB. Negative inotropic mediators released from the heart after myocardial ischaemia-reperfusion. *Cardiovasc Res.* (2002) 53:12–30. 10.1016/S0008-6363(01)00420-5 11744010

[65] Kojda G Laursen JB Ramasamy S Kent JD Kurz S Burchfield J Protein expression, vascular reactivity and soluble guanylate cyclase activity in mice lacking the endothelial cell nitric oxide synthase: contributions of NOS isoforms to blood pressure and heart rate control. *Cardiovasc Res.* (1999) 42:206–13. 10.1016/S0008-6363(98)00315-0 10435012

[66] Chandrasekar B Vemula K Surabhi RM Li-Weber M Owen-Schaub LB Jensen LE. Activation of intrinsic and extrinsic proapoptotic signaling pathways in interleukin-18-mediated human cardiac endothelial cell death. *J Biol Chem.* (2004) 279:20221–33. 10.1074/jbc.M313980200 14960579

[67] Rudolph JL Ramlawi B Kuchel GA McElhaney JE Xie D Sellke FW Chemokines are associated with delirium after cardiac surgery. *J Gerontol A Biol Sci Med Sci.* (2008) 63:184–9. 10.1093/gerona/63.2.184 18314455 PMC2735245

[68] Cerejeira J Firmino H Vaz-Serra A Mukaetova-Ladinska EB. The neuroinflammatory hypothesis of delirium. *Acta Neuropathol.* (2010) 119:737–54. 10.1007/s00401-010-0674-1 20309566

[69] Kok WF Koerts J Tucha O Scheeren TW Absalom AR. Neuronal damage biomarkers in the identification of patients at risk of long-term postoperative cognitive dysfunction after cardiac surgery. *Anaesthesia.* (2017) 72:359–69. 10.1111/anae.13712 27987229

[70] Shaw PJ Bates D Cartlidge NE French JM Heaviside D Julian DG Neurologic and neuropsychological morbidity following major surgery: comparison of coronary artery bypass and peripheral vascular surgery. *Stroke.* (1987) 18:700–7. 10.1161/01.STR.18.4.700 3496690

[71] Kennedy ED Choy KC Alston RP Chen S Farhan-Alanie MM Anderson J. Cognitive outcome after on- and off-pump coronary artery bypass grafting surgery: a systematic review and meta-analysis. *J Cardiothorac Vasc Anesth.* (2013) 27:253–65. 10.1053/j.jvca.2012.11.008 23507014

[72] Steinmetz J Rasmussen LS. Peri-operative cognitive dysfunction and protection. *Anaesthesia.* (2016) 71(Suppl 1):58–63. 10.1111/anae.13308 26620148

[73] Salameh A Dhein S Dähnert I Klein N. Neuroprotective strategies during cardiac surgery with cardiopulmonary bypass. *Int J Mol Sci.* (2016) 17:1945. 10.3390/ijms17111945 27879647 PMC5133939

[74] Patel N Minhas JS Chung EM. Intraoperative embolization and cognitive decline after cardiac surgery: a systematic review. *Semin Cardiothorac Vasc Anesth.* (2016) 20:225–31. 10.1177/1089253215626728 26783262

[75] Lamy A Devereaux PJ Prabhakaran D Taggart DP Hu S Paolasso E Effects of off-pump and on-pump coronary-artery bypass grafting at 1 year. *N Engl J Med.* (2013) 368:1179–88. 10.1056/NEJMoa1301228 23477676

[76] Berger M Terrando N Smith SK Browndyke JN Newman MF Mathew JP. Neurocognitive function after cardiac surgery: from phenotypes to mechanisms. *Anesthesiology.* (2018) 129:829–51. 10.1097/ALN.0000000000002194 29621031 PMC6148379

[77] Bhamidipati D Goldhammer JE Sperling MR Torjman MC McCarey MM Whellan DJ. Cognitive outcomes after coronary artery bypass grafting. *J Cardiothorac Vasc Anesth.* (2017) 31:707–18. 10.1053/j.jvca.2016.09.028 28094177

[78] Inouye SK Ferrucci L. Elucidating the pathophysiology of delirium and the interrelationship of delirium and dementia. *J Gerontol A Biol Sci Med Sci.* (2006) 61:1277–80. 10.1093/gerona/61.12.1277 17234820 PMC2645654

[79] Glumac S Kardum G Sodic L Supe-Domic D Karanovic N. Effects of dexamethasone on early cognitive decline after cardiac surgery: a randomised controlled trial. *Eur J Anaesthesiol.* (2017) 34:776–84. 10.1097/EJA.0000000000000647 28985195

[80] Gupta RK Patel AK Shah N Chaudhary AK Jha UK Yadav UC Oxidative stress and antioxidants in disease and cancer: a review. *Asian Pac J Cancer Prev.* (2014) 15:4405–9. 10.7314/APJCP.2014.15.11.4405 24969860

[81] Noctor G Lelarge-Trouverie C Mhamdi A. The metabolomics of oxidative stress. *Phytochemistry.* (2015) 112:33–53. 10.1016/j.phytochem.2014.09.002 25306398

[82] Nabeebaccus A Zhang M Shah AM. NADPH oxidases and cardiac remodelling. *Heart Fail Rev.* (2011) 16:5–12. 10.1007/s10741-010-9186-2 20658317

[83] Zhang YH Jin CZ Jang JH Wang Y. Molecular mechanisms of neuronal nitric oxide synthase in cardiac function and pathophysiology. *J Physiol.* (2014) 592:3189–200. 10.1113/jphysiol.2013.270306 24756636 PMC4146369

[84] Barzegar Amiri Olia M Schiesser CH Taylor MK. New reagents for detecting free radicals and oxidative stress. *Org Biomol Chem.* (2014) 12:6757–66. 10.1039/C4OB01172D 25053503

[85] Dias AE Melnikov P Cônsolo LZ. Oxidative stress in coronary artery bypass surgery. *Rev Bras Cir Cardiovasc.* (2015) 30:417–24. 10.5935/1678-9741.20150052 27163415 PMC4614924

[86] Ermak G Davies KJ. Calcium and oxidative stress: from cell signaling to cell death. *Mol Immunol.* (2002) 38:713–21. 10.1016/S0161-5890(01)00108-0 11841831

[87] Baufreton C Corbeau JJ Pinaud F. [Inflammatory response and haematological disorders in cardiac surgery: toward a more physiological cardiopulmonary bypass]. *Ann Fr Anesth Reanim.* (2006) 25:510–20. 10.1016/j.annfar.2005.12.002 16488106

[88] Dabbous A Kassas C Baraka A. The inflammatory response after cardiac surgery. *Middle East J Anaesthesiol.* (2003) 17:233–54. 14503124

[89] Kirklin JK McGiffin DC. Early complications following cardiac surgery. *Cardiovasc Clin.* (1987) 17:321–43. 3555815

[90] Ng CS Wan S. Limiting inflammatory response to cardiopulmonary bypass: pharmaceutical strategies. *Curr Opin Pharmacol.* (2012) 12:155–9. 10.1016/j.coph.2012.01.007 22305683

[91] Suleiman MS Zacharowski K Angelini GD. Inflammatory response and cardioprotection during open-heart surgery: the importance of anaesthetics. *Br J Pharmacol.* (2008) 153:21–33. 10.1038/sj.bjp.0707526 17952108 PMC2199383

[92] Vinten-Johansen J. Involvement of neutrophils in the pathogenesis of lethal myocardial reperfusion injury. *Cardiovasc Res.* (2004) 61:481–97. 10.1016/j.cardiores.2003.10.011 14962479

[93] Dambrova M Zuurbier CJ Borutaite V Liepinsh E Makrecka-Kuka M. Energy substrate metabolism and mitochondrial oxidative stress in cardiac ischemia/reperfusion injury. *Free Radic Biol Med.* (2021) 165:24–37. 10.1016/j.freeradbiomed.2021.01.036 33484825

[94] Bonventre JV Zuk A. Ischemic acute renal failure: an inflammatory disease? *Kidney Int.* (2004) 66:480–5. 10.1111/j.1523-1755.2004.761_2.x 15253693

[95] Ozbek E. Induction of oxidative stress in kidney. *Int J Nephrol.* (2012) 2012:465897. 10.1155/2012/465897 22577546 PMC3345218

[96] Devarajan P. Update on mechanisms of ischemic acute kidney injury. *J Am Soc Nephrol.* (2006) 17:1503–20. 10.1681/ASN.2006010017 16707563

[97] Massoth C Zarbock A. Diagnosis of cardiac surgery-associated acute kidney injury. *J Clin Med.* (2021) 10:3664. 10.3390/jcm10163664 34441960 PMC8397056

[98] Young IS. Measurement of total antioxidant capacity. *J Clin Pathol.* (2001) 54:339. 10.1136/jcp.54.5.339 11328830 PMC1731415

[99] Gariballa SE Hutchin TP Sinclair AJ. Antioxidant capacity after acute ischaemic stroke. *QJM.* (2002) 95:685–90. 10.1093/qjmed/95.10.685 12324641

[100] Yao JK Reddy R McElhinny LG van Kammen DP. Reduced status of plasma total antioxidant capacity in schizophrenia. *Schizophr Res.* (1998) 32:1–8. 10.1016/S0920-9964(98)00030-9 9690328

[101] Loh KP Huang SH De Silva R Tan BK Zhu YZ. Oxidative stress: apoptosis in neuronal injury. *Curr Alzheimer Res.* (2006) 3:327–37. 10.2174/156720506778249515 17017863

[102] Bhat AH Dar KB Anees S Zargar MA Masood A Sofi MA Oxidative stress, mitochondrial dysfunction and neurodegenerative diseases; a mechanistic insight. *Biomed Pharmacother.* (2015) 74:101–10. 10.1016/j.biopha.2015.07.025 26349970

[103] Aldecoa C Bettelli G Bilotta F Sanders RD Audisio R Borozdina A European Society of Anaesthesiology evidence-based and consensus-based guideline on postoperative delirium. *Eur J Anaesthesiol.* (2017) 34:192–214. 10.1097/EJA.0000000000000594 28187050

[104] Chung KS Lee JK Park JS Choi CH. Risk factors of delirium in patients undergoing total knee arthroplasty. *Arch Gerontol Geriatr.* (2015) 60:443–7. 10.1016/j.archger.2015.01.021 25704295

[105] Chen H Mo L Hu H Ou Y Luo J. Risk factors of postoperative delirium after cardiac surgery: a meta-analysis. *J Cardiothorac Surg.* (2021) 16:113. 10.1186/s13019-021-01496-w 33902644 PMC8072735

[106] Sanson G Khlopenyuk Y Milocco S Sartori M Dreas L Fabiani A. Delirium after cardiac surgery. Incidence, phenotypes, predisposing and precipitating risk factors, and effects. *Heart Lung.* (2018) 47:408–17. 10.1016/j.hrtlng.2018.04.005 29751986

[107] O’Neal JB Billings FTT Liu X Shotwell MS Liang Y Shah AS. Risk factors for delirium after cardiac surgery: a historical cohort study outlining the influence of cardiopulmonary bypass. *Can J Anaesth.* (2017) 64:1129–37. 10.1007/s12630-017-0938-5 28718100 PMC5693689

[108] Smith LW Dimsdale JE. Postcardiotomy delirium: conclusions after 25 years? *Am J Psychiatry.* (1989) 146:452–8. 10.1176/ajp.146.4.452 2929744

[109] Gaudino M Angelini GD Antoniades C Bakaeen F Benedetto U Calafiore AM Off-Pump coronary artery bypass grafting: 30 years of debate. *J Am Heart Assoc.* (2018) 7:e009934. 10.1161/JAHA.118.009934 30369328 PMC6201399

[110] Rudiger A Begdeda H Babic D Krüger B Seifert B Schubert M. Intra-operative events during cardiac surgery are risk factors for the development of delirium in the ICU. *Crit Care.* (2016) 20:264. 10.1186/s13054-016-1445-8 27544077 PMC4992555

[111] Guo Y Jia P Zhang J Wang X Jiang H Jiang W. Prevalence and risk factors of postoperative delirium in elderly hip fracture patients. *J Int Med Res.* (2016) 44:317–27. 10.1177/0300060515624936 26920926 PMC5580064

[112] Li HC Chen YS Chiu MJ Fu MC Huang GH Chen CC. Delirium, subsyndromal delirium, and cognitive changes in individuals undergoing elective coronary artery bypass graft surgery. *J Cardiovasc Nurs.* (2015) 30:340–5. 10.1097/JCN.0000000000000170 24978158

[113] Chan MT Cheng BC Lee TM Gin T. BIS-guided anesthesia decreases postoperative delirium and cognitive decline. *J Neurosurg Anesthesiol.* (2013) 25:33–42. 10.1097/ANA.0b013e3182712fba 23027226

[114] Leslie K Myles PS Forbes A Chan MT. The effect of bispectral index monitoring on long-term survival in the B-aware trial. *Anesth Analg.* (2010) 110:816–22. 10.1213/ANE.0b013e3181c3bfb2 19910621

[115] Fritz BA Kalarickal PL Maybrier HR Muench MR Dearth D Chen Y Intraoperative Electroencephalogram Suppression Predicts Postoperative Delirium. *Anesth Analg.* (2016) 122:234–42. 10.1213/ANE.0000000000000989 26418126 PMC4684753

[116] Pérez-Otal B Aragón-Benedí C Pascual-Bellosta A Ortega-Lucea S Martínez-Ubieto J Ramírez-Rodríguez JM. Neuromonitoring depth of anesthesia and its association with postoperative delirium. *Sci Rep.* (2022) 12:12703. 10.1038/s41598-022-16466-y 35882875 PMC9325758

[117] Tan MC Felde A Kuskowski M Ward H Kelly RF Adabag AS Incidence and predictors of post-cardiotomy delirium. *Am J Geriatr Psychiatry.* (2008) 16:575–83. 10.1097/JGP.0b013e318172b418 18591577

[118] Subramaniam B Shankar P Shaefi S Mueller A O’Gara B Banner-Goodspeed V. Effect of intravenous acetaminophen vs placebo combined with propofol or dexmedetomidine on postoperative delirium among older patients following cardiac surgery: the DEXACET Randomized Clinical Trial. *JAMA.* (2019) 321:686–96. 10.1001/jama.2019.0234 30778597 PMC6439609

[119] Jean YH Wen ZH Chang YC Hsieh SP Tang CC Wang YH Intra-articular injection of the cyclooxygenase-2 inhibitor parecoxib attenuates osteoarthritis progression in anterior cruciate ligament-transected knee in rats: role of excitatory amino acids. *Osteoarthr Cartil.* (2007) 15:638–45. 10.1016/j.joca.2006.11.008 17198754

[120] Laine L White WB Rostom A Hochberg M. COX-2 selective inhibitors in the treatment of osteoarthritis. *Semin Arthritis Rheum.* (2008) 38:165–87. 10.1016/j.semarthrit.2007.10.004 18177922

[121] Ye Z Wang N Xia P Wang E Yuan Y Guo Q. Delayed administration of parecoxib, a specific COX-2 inhibitor, attenuated postischemic neuronal apoptosis by phosphorylation Akt and GSK-3β. *Neurochem Res.* (2012) 37:321–9. 10.1007/s11064-011-0615-y 21964800

[122] Sironi L Cimino M Guerrini U Calvio AM Lodetti B Asdente M Treatment with statins after induction of focal ischemia in rats reduces the extent of brain damage. *Arterioscler Thromb Vasc Biol.* (2003) 23:322–7. 10.1161/01.ATV.0000044458.23905.3B 12588778

[123] Blanco-Colio LM Tuñón J Martín-Ventura JL Egido J. Anti-inflammatory and immunomodulatory effects of statins. *Kidney Int.* (2003) 63:12–23. 10.1046/j.1523-1755.2003.00744.x 12472764

[124] Stepień K Tomaszewski M Czuczwar SJ. Neuroprotective properties of statins. *Pharmacol Rep.* (2005) 57:561–9. 16227638

[125] Rezaie-Majd A Maca T Bucek RA Valent P Müller MR Husslein P. Simvastatin reduces expression of cytokines interleukin-6, interleukin-8, and monocyte chemoattractant protein-1 in circulating monocytes from hypercholesterolemic patients. *Arterioscler Thromb Vasc Biol.* (2002) 22:1194–9. 10.1161/01.ATV.0000022694.16328.CC 12117737

[126] Lim S Barter P. Antioxidant effects of statins in the management of cardiometabolic disorders. *J Atheroscler Thromb.* (2014) 21:997–1010. 10.5551/jat.24398 25132378

[127] Katznelson R Djaiani GN Borger MA Friedman Z Abbey SE Fedorko L Preoperative use of statins is associated with reduced early delirium rates after cardiac surgery. *Anesthesiology.* (2009) 110:67–73. 10.1097/ALN.0b013e318190b4d9 19104172

[128] Mathew JP Grocott HP McCurdy JR Ti LK Davis RD Laskowitz DT Preoperative statin therapy does not reduce cognitive dysfunction after cardiopulmonary bypass. *J Cardiothorac Vasc Anesth.* (2005) 19:294–9. 10.1053/j.jvca.2005.03.004 16130053

[129] Mariscalco G Mariani S Biancari F Banach M. Effects of statins on delirium following cardiac surgery - evidence from literature. *Psychiatr Pol.* (2015) 49:1359–70. 10.12740/PP/60139 26909407

[130] Kazmierski J Kowman M Banach M Pawelczyk T Okonski P Iwaszkiewicz A Preoperative predictors of delirium after cardiac surgery: a preliminary study. *Gen Hosp Psychiatry.* (2006) 28:536–8. 10.1016/j.genhosppsych.2006.08.007 17088170

[131] Koster S Hensens AG Schuurmans MJ van der Palen J. Risk factors of delirium after cardiac surgery: a systematic review. *Eur J Cardiovasc Nurs.* (2011) 10:197–204. 10.1016/j.ejcnurse.2010.09.001 20870463

[132] Koenig MA Grega MA Bailey MM Pham LD Zeger SL Baumgartner WA Statin use and neurologic morbidity after coronary artery bypass grafting: A cohort study. *Neurology.* (2009) 73:2099–106. 10.1212/WNL.0b013e3181c677f6 19907012 PMC2790220

[133] Ford AH Almeida OP. Pharmacological interventions for preventing delirium in the elderly. *Maturitas.* (2015) 81:287–92. 10.1016/j.maturitas.2015.03.024 25890587

[134] Reiter RJ Mayo JC Tan DX Sainz RM Alatorre-Jimenez M Qin L. Melatonin as an antioxidant: under promises but over delivers. *J Pineal Res.* (2016) 61:253–78. 10.1111/jpi.12360 27500468

[135] Tan DX Manchester LC Qin L Reiter RJ. Melatonin: A Mitochondrial Targeting Molecule Involving Mitochondrial Protection and Dynamics. *Int J Mol Sci.* (2016) 17:2124. 10.3390/ijms17122124 27999288 PMC5187924

[136] Nabavi SM Nabavi SF Sureda A Xiao J Dehpour AR Shirooie S Anti-inflammatory effects of Melatonin: a mechanistic review. *Crit Rev Food Sci Nutr.* (2019) 59(Suppl. 1):S4–16. 10.1080/10408398.2018.1487927 29902071

[137] El-Shenawy SM Abdel-Salam OM Baiuomy AR El-Batran S Arbid MS. Studies on the anti-inflammatory and anti-nociceptive effects of melatonin in the rat. *Pharmacol Res.* (2002) 46:235–43. 10.1016/S1043-6618(02)00094-4 12220966

[138] Carrasco C Marchena AM Holguín-Arévalo MS Martín-Partido G Rodríguez AB Paredes SD. Anti-inflammatory effects of melatonin in a rat model of caerulein-induced acute pancreatitis. *Cell Biochem Funct.* (2013) 31:585–90. 10.1002/cbf.2942 24779037

[139] Shigeta H Yasui A Nimura Y Machida N Kageyama M Miura M Postoperative delirium and melatonin levels in elderly patients. *Am J Surg.* (2001) 182:449–54. 10.1016/S0002-9610(01)00761-9 11754849

[140] Asleson DR Chiu AW. Melatonin for delirium prevention in acute medically ill, and perioperative geriatric patients. *Aging Med.* (2020) 3:132–7. 10.1002/agm2.12112 32666028 PMC7338699

[141] Guler G Akin A Tosun Z Eskitascoglu E Mizrak A Boyaci A. Single-dose dexmedetomidine attenuates airway and circulatory reflexes during extubation. *Acta Anaesthesiol Scand.* (2005) 49:1088–91. 10.1111/j.1399-6576.2005.00780.x 16095449

[142] Zeng X Wang H Xing X Wang Q Li W. Dexmedetomidine protects against transient global cerebral ischemia/reperfusion induced oxidative stress and inflammation in diabetic rats. *PLoS One.* (2016) 11:e0151620. 10.1371/journal.pone.0151620 26982373 PMC4794239

[143] Xianbao L Hong Z Xu Z Chunfang Z Dunjin C. Dexmedetomidine reduced cytokine release during postpartum bleeding-induced multiple organ dysfunction syndrome in rats. *Mediators Inflamm.* (2013) 2013:627831. 10.1155/2013/627831 23840096 PMC3693180

[144] Cho JS Shim JK Soh S Kim MK Kwak YL. Perioperative dexmedetomidine reduces the incidence and severity of acute kidney injury following valvular heart surgery. *Kidney Int.* (2016) 89:693–700. 10.1038/ki.2015.306 26444030

[145] Wu J Vogel T Gao X Lin B Kulwin C Chen J. Neuroprotective effect of dexmedetomidine in a murine model of traumatic brain injury. *Sci Rep.* (2018) 8:4935. 10.1038/s41598-018-23003-3 29563509 PMC5862953

[146] Hu J Vacas S Feng X Lutrin D Uchida Y Lai IK Dexmedetomidine prevents cognitive decline by enhancing resolution of high mobility group box 1 protein-induced inflammation through a vagomimetic action in mice. *Anesthesiology.* (2018) 128:921–31. 10.1097/ALN.0000000000002038 29252509 PMC6445386

[147] Yang W Kong LS Zhu XX Wang RX Liu Y Chen LR. Effect of dexmedetomidine on postoperative cognitive dysfunction and inflammation in patients after general anaesthesia: A PRISMA-compliant systematic review and meta-analysis. *Medicine.* (2019) 98:e15383. 10.1097/MD.0000000000015383 31045788 PMC6504304

